# Transcription Factor DLX5 Promotes Hair Follicle Stem Cell Differentiation by Regulating the c-MYC/microRNA-29c-3p/NSD1 Axis

**DOI:** 10.3389/fcell.2021.554831

**Published:** 2021-07-15

**Authors:** Bojie Lin, Jiangying Zhu, Guoqian Yin, Mingde Liao, Guanyu Lin, Yuyong Yan, Dan Huang, Siding Lu

**Affiliations:** ^1^Department of Plastic and Aesthetic Surgery, The First Affiliated Hospital of Guangxi Medical University, Nanning, China; ^2^Academy of Humanities and Social Sciences, Guangxi Medical University, Nanning, China

**Keywords:** DLX5, c-Myc, microRNA-29c-3p, NSD1, Hair follicle stem cells

## Abstract

**Introduction:**

Adult stem cell function has been one of the most intensively explored areas of biological and biomedical research, with hair follicle stem cells serving as one of the best model systems. This study explored the role of the transcription factor DLX5 in regulating hair follicle stem cell (HFSC) differentiation.

**Methods:**

HFSCs were isolated, characterized, and assessed for their expression of DLX5, c-MYC, NSD1, and miR-29c-3p using RT-qPCR, Western blot analysis, or immunofluorescence. Next, the ability of HFSCs to proliferate as well as differentiate into either sebaceous gland cells or epidermal cells was determined. The binding of DLX5 to the c-MYC promoter region, the binding of c-MYC to the miR-29c-3p promoter region, and the binding of miR-29c-3p to the 3′-UTR of NSD1 mRNA were verified by luciferase activity assay and ChIP experiments.

**Results:**

DLX5 was highly expressed in differentiated HFSCs. DLX5 transcriptionally activated c-MYC expression to induce HFSC differentiation. c-MYC was able to bind the miR-29c-3p promoter and thus suppressed its expression. Without miR-29c-3p mediated suppression, NSD1 was then able to promote HFSC differentiation. These *in vitro* experiments suggested that DLX5 could promote HFSC differentiation via the regulation of the c-MYC/miR-29c-3p/NSD1 axis.

**Discussion:**

This study demonstrates that DLX5 promotes HFSC differentiation by modulating the c-MYC/miR-29c-3p/NSD1 axis and identifies a new mechanism regulating HFSC differentiation.

## Introduction

Hair follicle stem cells (HFSCs) give rise to both hair follicle and epidermal cells (Taylor et al., 2000). They are positioned in discrete compartments or niches at the bottom of the follicle and can be easily isolated (Waters et al., 2007). HFSCs possess features common to all adult stem cells, including high proliferative ability, the ability to self-renew and the ability to differentiate into multiple lineages (Wang et al., 2013; Shen et al., 2017). Their capacity to proliferate quickly makes HFSCs a potential source of stem cells used in cell therapies (Najafzadeh et al., 2015a). In addition, HFSCs have the ability to differentiate into multiple cell lineages, including hair follicle cells, keratinocyte cells, endothelial cells, sebaceous glands cells, and even neural cells (Najafzadeh et al., 2015b; Zhang et al., 2016; Joulai Veijouyeh et al., 2017; Quan et al., 2017). However, promoting differentiation into these lineages *in vitro* and *in vivo* has been difficult.

Distal-less homeobox 5 (DLX5) is a homeodomain transcription factor that is encoded by a mammalian homolog a DLX gene that regulates the development of various cell types (Heo et al., 2017). The expression of DLX5 is essential for the cephalic neural crest cell-mesoderm interactions that contribute to myogenic determination, patterning and differentiation (Heude et al., 2010). Li et al. (2018) revealed that miR-339-5p negatively regulated loureirin A-induced differentiation of HFSCs by binding to DLX5 leading to inactivation of the Wnt/β-catenin signaling pathway. Therefore, we aim to further clarify the mechanism of DLX5 in regulating HFSC differentiation. In terms of the potential mechanism of DLX5 for expression of target gene expression, evidence suggests that DLX5 transcriptionally activates target genes by directly binding to respective promoter (Hassan et al., 2004; Kim et al., 2004). c-MYC is an essential member of MYC gene family, and is known for its role in regulating unlimited cell proliferation and stem cell differentiation (Arnold and Watt, 2001). A prior study has indicated that the MYC promoter was specifically activated by DLX5 and that its promoter contains two DLX5 binding sites required for its transcriptional activation (Xu and Testa, 2009). Therefore, in this work, we aim to explore whether DLX5-mediated regulation of c-MYC influences the differentiation of HFSCs. MYC gene has been implicated in the modulation of miRNA expression usually resulting in widespread miRNA repression (Zhang X. et al., 2012). A recent work implicated MYC in regulation of miR-29 expression and that HDAC, with MYC, is important in miR-29b silencing in acute myeloid leukemia cells (Liu et al., 2010). Treatment with transforming growth factor-β1 resulted in differentiation into smooth muscle cells and a decrease in miR-29c expression (Wang et al., 2016). Evidence also suggests that c-MYC binds to the promoter of miR-29c suppressing its transcription (Chang et al., 2008). Therefore, we investigated whether c-MYC regulates HFSC differentiation by controlling miR-29c-3p expression. Nuclear receptor-binding SET-domain-containing protein (NSD1) is a mouse nuclear protein containing proline-tryptophan-tryptophan-proline (PWWP), su(var)3-9, enhancer-of-zeste, trithorax (SET) domain, as well as plant homeodomain protein (PHD)-finger domains (Kurotaki et al., 2001). NSD1 is a developmental regulatory protein that possesses cellular functions, which are essential for early post-implantation mouse development (Rayasam et al., 2003). Based on aforementioned evidence, we speculated that DLX, c-MYC, miR-29c-3p, and NSD1 might interact during HFSC differentiation.

## Materials and Methods

### Ethics Statement

The animal experiments were carried out according to the Guide for the Care and Use of Laboratory Animals published by the United States National Institutes of Health. All the experiments were approved by the Animal Ethics Committee of the First Affiliated Hospital of Guangxi Medical University.

### Experimental Animals

Ten specific pathogen-free (SPF) Sprague-Dawley (SD) suckling rats, 8–9 days after birth, regardless of gender, were used for HFSC isolation. Fifteen SPF 4-week-old nude mice were used for *in vivo* transplantation experiments. All experimental animals were purchased from Shanghai SLAC Laboratory Animal Co., Ltd. (Shanghai, China).

### Isolation and Culture of HFSCs

Sprague-Dawley suckling rats (8–9 days), with an average weight of about 8.2 g, were euthanized to cut off the tentacles. The skin and subcutaneous tissues of the tentacles after disinfection were removed, and the subcutaneous tissues were clipped under a stereomicroscope. Each complete single hair follicle was dissected and the upper hair shaft and hair bulb were cut off. The collected hair follicle tissues were placed in 5 mL Dispase II (5 mg/mL) solution, dissociated at 100 rpm at 37°C for 2 h and centrifuged at 2,000 rpm for 2 min to remove the supernatant. After removing Dispase II, cells were resuspended in 3 mL of 0.125% trypsin-0.01% ethylene diamine tetraacetic acid solution and centrifuged at 100 rpm at 37°C for 30 min until the hair follicle tissues were broken down into floccule, followed by termination of the dissociation with medium containing 10% fetal bovine serum (FBS). The detached cell solution was filtered through a 200-mesh sieve to remove any undigested tissue mass, the flow-through solution was collected and transferred into a 10 mL centrifuge tube. The cells were then supplemented with culture medium and counted. After HFSCs were centrifuged, the supernatant was removed, and then the cells were resuspended in 30 μL of medium and labeled with 10 μL of CD34 monoclonal antibody in dark at 4°C for 30 min. Subsequently, the HFSCs were centrifuged at 1,200 rpm for 3 min, resuspended with 30 μL buffer, and labeled with the addition of 20 μL anti-PE magnetic beads without exposure to light at 4°C for 20 min. After being washed with 5 mL buffer, HFSCs were centrifuged at 1,200 rpm for 3 min and resuspended with 500 μL buffer. Next, the column was removed from the magnetic field and washed with 500 μL buffer. CD34 + cells were pushed out using the plunger. Cells were seeded at 5 × 10^4^ cells/mL in a 25 mL culture flask coated with type IV collagen, and cultured in an incubator at 37°C with 5% CO_2_. The cells would be used once they reached 80% confluence. The purity of HFSCs was detected using the flow cytometer.

### Immunofluorescence

Hair follicle stem cells were characterized after they were fixed with 4% paraformaldehyde for 30 min, blocked with 1% bovine serum albumin for 1 h and incubated with the primary antibody against K15 (1:100, Abcam, Cambridge, United States) and CD34 (1:200, Abcam, Cambridge, United States). Next, the cells were incubated with fluorescein isothiocyanate (FITC)-marked IgG and CY3-marked IgG secondary antibodies (Santa Cruz Biotechnology, Santa Cruz, CA, United States) for 1 h. The nucleus was stained with 4′,6-diamidino-2-phenylindole (DAPI) and cells were observed under a microscope.

Fifteen BALB/c nude mice (4–5 weeks old, weighing 17–22 g) were purchased from Vital River Lab Animal Technology Co., Ltd. (Beijing, China) and reared in SPF animal facility (National Research Council, 2011). Skin tissues from nude mice were fixed with 4% paraformaldehyde overnight, washed with 0.01 M phosphate buffered saline (PBS) three times, and blocked with 10% goat serum at room temperature for 30 min. Next, the tissues were incubated with the primary antibodies DLX5 (1:100, ab239987, Abcam, Cambridge, United States), c-MYC (1:100, ab223913, Abcam, Cambridge, United States), and NSD1 (1:200, ab226247, Abcam, Cambridge, United States) overnight at 4°C and then incubated with secondary antibody and DAPI at room temperature without exposure to light for 1 h, followed by fixation with glycerol. The cells were examined using confocal laser microscopy and the images were captured using a scanning microscope (LSM, FV1000; Olympus Corporation, Tokyo, Japan). Immunofluorescence intensity was quantified by the Image-Pro Plus 6.0 software (Media Cybernetics, Silver Spring, MD, United States) (Min et al., 2018).

### Differentiation of HFSCs Into Sebaceous Gland Cells and Oil Red O Staining

Hair follicle stem cells were collected by trypsinization at about 80% confluence and seeded into a 35 mm petri dish at a density of 8 × 10^3^ cells/cm^2^. When the cell confluence reached 60%, the cells were given adipogenic culture medium (experimental group). Additionally, another dish of cells was supplemented with normal culture medium for culture (control group). The media for both the experimental group and the control group were then replaced every 3 days, and the cells were observed under a microscope after 2 weeks. Once a large number of transparent lipid droplets had formed in the monolayer of cells, oil red O staining was performed.

After removing the adipogenic induction medium, the cells were fixed with 4% paraformaldehyde solution for 30 min, and supplemented with 60% isopropanol to wash off excess paraformaldehyde fixation solution. Finally, the cells were stained for 5–10 min with freshly prepared 0.6% oil red O working solution filtered through filter paper and then observed under a microscope. If the transparent lipid droplets were stained well, the cells were treated with 60% isopropyl alcohol to decolorize the background color, and then observed under the microscope.

### Differentiation of HFSCs Into Epidermal Cells

Hair follicle stem cells at passage two were seeded at a density of 5 × 10^4^ cells/well into a 6-well plate pre-coated with type IV collagen, incubated with keratinocyte induction medium, and placed at 37°C with 5% CO_2_ and 95% humidity. The cell morphology and cell growth status were observed under a microscope every day. The fluid was changed every other day until the cell confluence reached 80%. After counting, the cells were sub-cultured at the same density and continued to be incubated in the induction medium until the cells were confluent again.

### Flow Cytometry

Cells were characterized by flow cytometry after detachment with trypsin at 37°C for 5–10 min. After detachment, trypsin was neutralized by the addition of medium containing FBS. Next, the cells were centrifuged at 1,000 rpm for 5 min and then the supernatant was removed. Again, the cells were fixed with 4% paraformaldehyde for 30 min and centrifuged at 1,000 rpm for 5 min and the supernatant was discarded. Subsequently, the cells were incubated with 0.1% Triton X-100 for 10 min, and cell density was adjusted to 1 × 10^6^ cells/mL. Cells were then incubated with 200 μL of diluted fluorescently labeled antibody (APC-labeled mouse anti-human CK10 monoclonal antibody, PE-labeled mouse anti-human CD200 monoclonal antibody, and FITC-labeled mouse anti-human EMA monoclonal antibody) on ice for 30 min, followed by centrifugation at 1,000 rpm for 5 min. The supernatant containing excess unbound antibody was removed and cells were subsequently resuspended in 500 μL of cold PBS and then processed at 4°C in the dark by a flow cytometer.

### Cell Counting Kit-8 (CCK-8) Assay

Hair follicle stem cells at 80% confluence were rinsed twice with PBS, and detached with 0.25% trypsin to prepare for single cell suspensions. After counting, 3 × 10^3^ to 6 × 10^3^ cells per well were seeded in a 96-well plate with a volume of 200 μL per well and incubated at 37°C with 5% CO_2_. The cells were cultured for 24, 48, and 72 h, respectively. Each well was treated with 10 μL CCK-8 solution (Sigma-Aldrich, St. Louis, MO, United States) for 2-h culture, and the optical density (OD) value at 450 nm was detected by a microplate reader (NYW-96M, Beijing Nuoyawei Instrument Co., Ltd., Beijing, China).

### Reverse Transcription Quantitative Polymerase Chain Reaction (RT-qPCR)

Total RNA was extracted from tissues and cells using TRIzol reagents (Invitrogen, Carlsbad, CA, United States), and the nanodrop2000 micro-ultraviolet spectrophotometer (1011U, NanoDrop Technologies, Inc., Rockland, ME, United States) was used to detect the total RNA concentration and purity. Reverse transcription of RNA into complementary DNA (cDNA) was performed according to the instructions of the TaqMan MicroRNA Assays Reverse Transcription primer (4427975, Applied Biosystems, Foster City, CA, United States)/PrimeScript RT reagent Kit (RR047A, TaKaRa, Japan). The primer sequences for DLX5, c-MYC, miR-29c-3p and NSD1 were synthesized by TaKaRa company (Supplementary Table 1). ABI 7500 quantitative PCR instrument (Applied Biosystems) was utilized for RT-qPCR detection. Glyceraldehyde-3-phosphate dehydrogenase (GAPDH) was used as the internal reference, and the 2^−ΔΔCT^ method was used to calculate the relative mRNA expression level of each target gene.

### Cell Culture and Grouping

Hair follicle stem cells were cultured with RPMI 1640 medium supplemented with 10% FBS and placed in an incubator at 37°C with 5% CO_2_. After the cells adhered to the wall, they were detached with 0.25% trypsin (Hyclone, Logan, UT, United States). Only cells in the logarithmic growth phase were used for experiments.

Hair follicle stem cells were transfected with expression vector containing the DLX5 gene (oe-DLX5), shRNA against DLX5 (sh-DLX5), oe-c-MYC, sh-c-MYC, miR-29c-3p mimic, miR-29c-3p inhibitor, oe-NSD1 alone or in combination using lipofectamine 2000 reagents (Invitrogen) as per the manufacturer’s guidance. These plasmids, mimic and inhibitor were purchased and synthesized by Sino Biological (Beijing, China).

### Western Blot Analysis

Hair follicle stem cells from each group were lysed in radioimmunoprecipitation assay lysis buffer (Beyotime Biotechnology, Shanghai, China) for 5 min on ice, and centrifuged at 14,000 rpm at 4°C and the supernatant was collected. The protein concentration for each sample was measured using the bicinchoninic acid (Pierce, Rockford, IL, United States) method. Sodium dodecyl sulfate-polyacrylamide gel electrophoresis (SDS-PAGE) was prepared for electrophoresis at concentrations of 4 and 10% and samples were separated by SDS-PAGE and subsequently transferred onto a polyvinylidene difluoride membrane. Next the membrane was blocked with 5% skimmed milk powder for 1 h at room temperature, and then incubated with the primary antibody (anti-rabbit DLX5, 1: 1,000-1: 10,000; anti-rabbit c-MYC, 1: 1,000; anti-rabbit NSD1, 1: 1,000; Abcam) at 4°C overnight. After being washed with phosphate buffered saline tween (PBST), the membrane was incubated with the secondary antibody, horseradish peroxidase-labeled goat anti-rabbit IgG (Santa Cruz Biotechnology), for 1 h at room temperature. Bands were detected using the enhanced chemiluminescence reaction solution (Thermo Fisher, United States) at room temperature and observed for color development, then developed and fixed (Bio-Rad ChemiDoc^TM^ imaging system). GAPDH (anti-mouse, Santa Cruz Biotechnology) was used as an internal reference, and the band intensity was compared using ImageJ2x software.

### Luciferase Activity Assay

A fragment of c-MYC promoter containing the predicted DLX5 binding site and a fragment in which the binding site was mutated were inserted into the luciferase reporter vectors as reporter plasmids, namely c-MYC promoter-wild type (WT) and c-MYC promoter-mutant type (MUT), and co-transfected into 293T cells (Oulu Biotechnology, Guangzhou, China) with either oe-NC or oe-DLX5 plasmids. A fragment of the miR-29c-3p promoter containing the putative c-MYC binding site and a fragment of the miR-29c-3p promoter in which the c-MYC binding site had been mutated were inserted into luciferase reporter vectors as reporter plasmids: miR-29c-3p promoter-WT and miR-29c-3p promoter-MUT, and co-transfected with oe-NC or oe-c-MYC plasmid Finally, a fragment from NSD1 mRNA containing the predicted miR-29c-3p binding site and a fragment in which the miR-29c-3p binding site was mutated were inserted into luciferase reporter vectors as reporter plasmids: NSD1-WT and NSD1-MUT and were con-transfected with NC or miR-29c-3p mimic to determine whether miR-29c-3p could bind to the 3′-UTR of NSD1 mRNA. Cells were collected and lysed 48 h after transfection. The luciferase reporter gene assay was performed using a dual luciferase reporter gene analysis system (Promega, Madison, WI, United States) and the luciferase detection kit (K801-200, BioVision, Palo Alto, CA, United States). Renilla Luciferase was used as an internal reference.

### Chromatin Immunoprecipitation (ChIP)

Hair follicle stem cells before and after differentiation were cultured, and cells were fixed with 1% formaldehyde solution once the cell density reached 1 × 10^6^ cells/10 cm culture dish, and then placed on ice for 5 min to terminate the fixation. After washing, the cells were prepared for cell precipitation by detachment and centrifugation. The cells were suspended in 200 μL sodium dodecyl sulfate lysis buffer, placed on ice for 10 min to allow for cross-linking, and subsequently ultrasonicated to cut chromatin DNA while on ice. After diluting the supernatant with ChIP dilution buffer containing a protease inhibitor, the blocking solution was added and incubated at 4°C for 30 min. A small amount of supernatant was saved to serve as input, and the remaining supernatant was treated with DLX5 antibody (anti-rabbit, Abcam), or c-MYC antibody (anti-rabbit, Abcam), and the other part was added with IgG (anti-rabbit, Abcam) and incubated at 4°C overnight. Cross-linked agar was added and incubated for 1 h at 4°C to collect the antibody/transcription factor complex. The cell precipitation with full washing was eluted with elution buffer. The eluted supernatant and input DNA were treated with 20 μL of 5 mol/L NaCl to disrupt cross-linking. Proteinase K digestion was performed to remove protein and DNA was purified and recovered. Using the recovered DNA as a template, the relative expression of c-MYC (after addition of DLX5 antibody) or miR-29c-3p (after addition of c-MYC antibody) was detected using RT-qPCR.

### Fluorescence *in situ* Hybridization (FISH)

FISH was performed using the previously described protocol (Shi et al., 2018; Moldovan et al., 2019). The digoxigenin-labeled miR-29c-3p was hybridized with the disturbed LNA probe (Exiqon) at 61°C. The signal *in situ* was stained with digoxin-labeled anti-digoxin AP antibody (Roche Diagnostics GmbH, Mannheim, Germany), and developed with BM purple substrate (Roche). Fluorescence intensity was quantified by the Image Pro Plus 6.0 software (Media Cybernetics).

### Xenograft Tumor in Nude Mice

Fifteen BALB/c nude mice aged 4–5 weeks were randomly classified into 3 groups, 5 in each group. Under sterile conditions, the mice were routinely sterilized and anesthetized by intraperitoneal injection with 10% pentobarbital sodium at a dose of 50 mg/kg. A 2.5 mm full-thickness skin wound was created on the back with a skin biopsy puncher. Then 1 × 10^7^ dermal cells were mixed with 2 × 10^7^ HFSCs, and the mixture was added with 10 mL of Matrigel (BD Biosciences, San Jose, CA, United States) and mixed. After incubation at 37°C for 30 min, the cell-Matrigel mixture was implanted into the excised wound, which was covered with a self-adhesive elastic bandage. After 3 weeks, the mice were euthanized, after which the number of hair follicles was counted under an anatomical microscope, and the wound tissue samples were collected for histological analysis.

### Statistical Analysis

SPSS 21.0 (IBM, Armonk, New York, United States) statistical software was used for statistical analysis. Measurement data were expressed as mean ± standard deviation, and the two sets of data in an unpaired design were compared by unpaired *t* test. Data comparison among multiple groups was performed using one-way analysis of variance (ANOVA) and Tukey’s test. Data comparison among multiple groups at different time points was performed using two-way ANOVA followed by a Bonferroni *post hoc* test. *p* < 0.05 indicated a statistically significant difference.

## Results

### DLX5 Is Highly Expressed in Differentiated HFSCs and Promotes Cell Differentiation

**FIGURE 1:**
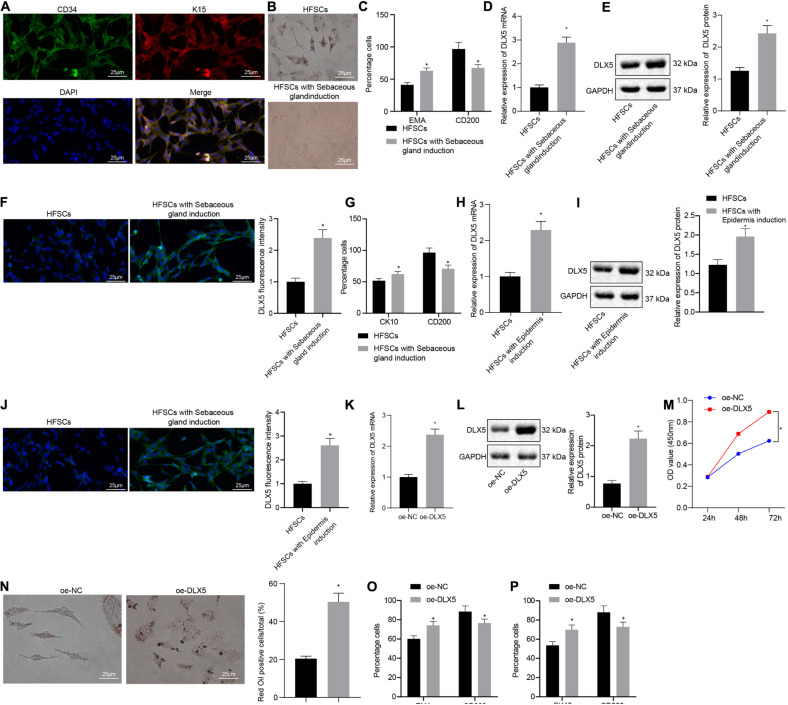
Overexpression of DLX5 in HFSCs promotes HFSC differentiation. **(A)** HFSC markers CD34 and K15 were identified by immunofluorescence assay for HFSC isolation (scale bar = 25 μm). **(B)** Oil red O staining before and after differentiation of HFSCs into sebaceous gland cells (scale bar = 25 μm). *p < 0.05 compared with HFSCs. **(C)** Flow cytometry detected the expression of EMA, a lipid droplet marker, and CD200, a specific marker of HFSCs, before and after HFSCs were differentiated into sebaceous gland cells. *p < 0.05 compared with HFSCs. **(D)** DLX5 mRNA expression in HFSCs after differentiation into sebaceous gland cells determined by RT-qPCR. *p < 0.05 compared with HFSCs. **(E)** DLX5 protein level in HFSCs after differentiation into sebaceous gland cells determined by Western blot analysis. *p < 0.05 compared with HFSCs. **(F)** DLX5 fluorescence intensity in HFSCs after differentiation into sebaceous gland cells determined by immunofluorescence assay. *p < 0.05 compared with HFSCs. **(G)** Detection of keratin CK10 and CD200 expression using flow cytometry, before and after the differentiation of HFSC cells into epidermal cells. *p < 0.05 compared with HFSCs. **(H)** DLX5 mRNA expression in HFSCs after differentiation into epidermal cells measured using RT-qPCR. *p < 0.05 compared with HFSCs. **(I)** DLX5 protein level in HFSCs after differentiation into epidermal cells measured using Western blot analysis. *p < 0.05 compared with HFSCs. **(J)** DLX5 fluorescence intensity in HFSCs after differentiation into epidermal cells measured using immunofluorescence assay. *p < 0.05 compared with HFSCs. **(K)** DLX5 mRNA expression in HFSCs determined by RT-qPCR. *p < 0.05 compared with treatment of oe-NC. **(L)** DLX5 protein expression in HFSCs determined by Western blot analysis. *p < 0.05 compared with treatment of oe-NC. **(M)** CCK-8 assay results of the proliferation rate in HFSCs after DLX5 overexpression. *p < 0.05 compared with treatment of oe-NC. **(N)** Oil red O staining before and after differentiation of HFSCs into sebaceous gland cells (scale bar = 25 μm). *p < 0.05 compared with treatment of oe-NC. **(O)** Flow cytometry detection of EMA and CD200 expression. *p < 0.05 compared with treatment of oe-NC. **(P)** Flow cytometry detection on the keratin CK10, and CD200 expression. *p < 0.05 compared with treatment of oe-NC. The two groups were compared by unpaired *t* test. Data among multiple groups at different time points were compared using two-way ANOVA and Bonferroni test. The experiment was repeated three times.

Immunofluorescence assay was conducted to detect HFSC markers CD34 and K15 (Figure 1A). After HFSCs were induced to differentiate into sebaceous cells, a large number of clear lipid droplets had formed in the cells, and oil red O staining was positive. No lipid droplet formation was observed in the untreated group (Figure 1B). Flow cytometry detected a significant increase in the number of lipid droplet marker, EMA-positive cells and a decrease in the number of HFSC-specific markers CD200-positive cells after induction (Figure 1C), indicating that HFSCs had successfully differentiated into sebaceous gland cells. Moreover, the results of RT-qPCR, Western blot analysis and immunofluorescence assay suggested that the expression of DLX5 mRNA and protein was elevated in HFSCs differentiated cells compared with the undifferentiated control group (Figures 1D–F). Flow cytometric data indicated that the number of CK10-positive cells in epidermal differentiation cells was significantly increased, and the number of CD200-positive cells was decreased (Figure 1G). Meanwhile, the results of RT-qPCR, Western blot analysis and immunofluorescence assay suggested that the expression levels of DLX5 mRNA and protein were increased in HFSCs differentiated cells compared with the undifferentiated control group (Figures 1H–J). These results indicated that DLX5 was highly expressed in differentiated HFSCs. In order to explore the function of DLX5 protein, we constructed DLX5-overexpressing HFSCs for the following experiments. The results of RT-qPCR and Western blot analysis showed that DLX5 overexpression caused an upward trend in the DLX5 mRNA and protein expression (Figures 1K,L). Subsequently, results revealed that DLX5 overexpression resulted in enhanced HFSC viability (Figure 1M), more oil red O-stained cells (Figure 1N), elevated sebaceous gland cell differentiation (Figure 1O), and enhanced epidermal cell differentiation (Figure 1P). The above results indicate that overexpression of DLX5 in HFSCs can promote HFSC differentiation.

### DLX5 Transcriptionally Activates c-MYC Expression to Promote HFSC Differentiation

**FIGURE 2:**
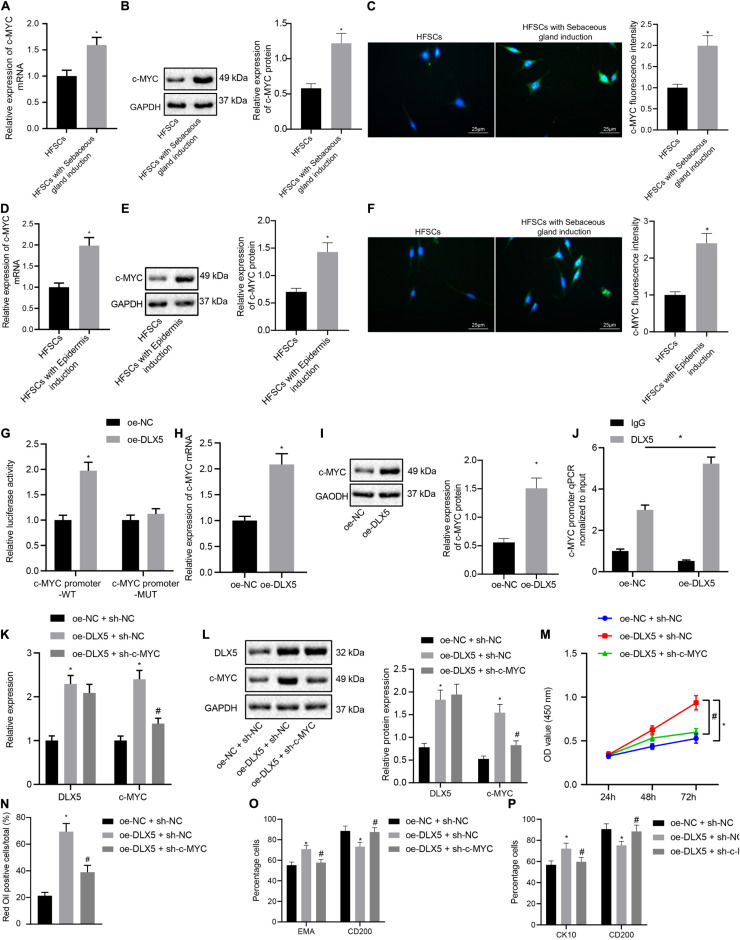
DLX5 promotes the differentiation of HFSCs by up-regulating the expression of c-MYC. **(A)** c-MYC mRNA expression in HFSCs after differentiation into sebaceous gland cells as determined by RT-qPC. *p < 0.05 compared with HFSCs. **(B)** c-MYC protein level in HFSCs after differentiation into sebaceous gland cells as determined by Western blot analysis. *p < 0.05 compared with HFSCs. **(C)** c-MYC fluorescence intensity in HFSCs after differentiation into sebaceous gland cells as determined by immunofluorescence assay. *p < 0.05 compared with HFSCs. **(D)** c-MYC mRNA expression in HFSCs after differentiation into epidermal cells as determined by RT-qPCR. *p < 0.05 compared with HFSCs. **(E)** c-MYC protein level in HFSCs after differentiation into epidermal cells as determined by Western blot analysis. *p < 0.05 compared with HFSCs. **(F)** c-MYC fluorescence intensity in HFSCs after differentiation into epidermal cells as determined by immunofluorescence assay. *p < 0.05 compared with HFSCs. **(G)** Luciferase activity assay was used to detect DLX5 binding of the c-MYC promoter. *p < 0.05 compared with treatment of oe-NC. **(H)** c-MYC mRNA expression in HFSCs treated with oe-DLX5 as determined by RT-qPCR. *p < 0.05 compared with treatment of oe-NC. **(I)** c-MYC protein level in HFSCs treated with oe-DLX5 as determined by Western blot analysis. *p < 0.05 compared with treatment of oe-NC. **(J)** ChIP experiment used to verify whether DLX5 could bind with c-MYC promoter. **(K)** DLX5 and c-MYC mRNA expressions in HFSCs determined by RT-qPCR.*p < 0.05 compared with treatment of oe-NC + sh-NC. #*p* < 0.05 compared with treatment of oe-DLX5 + sh-NC. **(L)** DLX5 and c-MYC protein levels in HFSCs determined by Western blot analysis. *p < 0.05 compared with treatment of oe-NC + sh-NC. #*p* < 0.05 compared with treatment of oe-DLX5 + sh-NC. **(M)** CCK-8 assay used to detect the proliferation rate of HFSCs. *p < 0.05 compared with treatment of oe-NC + sh-NC. #*p* < 0.05 compared with treatment of oe-DLX5 + sh-NC. **(N)** Oil red O staining before and after the differentiation of HFSCs into sebaceous gland cells.*p < 0.05 compared with treatment of oe-NC + sh-NC. #*p* < 0.05 compared with treatment of oe-DLX5 + sh-NC. **(O)** Detection of EMA and CD200 expression before and after the differentiation HFSCs into sebaceous gland cells using flow cytometry. *p < 0.05 compared with treatment of oe-NC + sh-NC. #*p* < 0.05 compared with treatment of oe-DLX5 + sh-NC. **(P)** Detection of keratin CK10 and CD200 expression before and after the differentiation of HFSCs into epidermal cells using flow cytometry. *p < 0.05 compared with treatment of oe-NC + sh-NC. #*p* < 0.05 compared with treatment of oe-DLX5 + sh-NC. Data are expressed as mean ± standard deviation, and comparisons between two groups were made using an unpaired *t* test. Comparisons among multiple groups were performed using one-way ANOVA and Tukey’s test. Comparisons among multiple groups at different time points were performed using two-way ANOVA and Bonferroni test. The experiment was repeated three times.

We next investigated whether DLX5 affected the differentiation of HFSCs by regulating c-MYC expression. The results of RT-qPCR, Western blot analysis and immunofluorescence assay indicated that the expression levels of c-MYC mRNA and protein were elevated after HFSCs differentiated into sebaceous gland cells or epidermal cells (Figures 2A–F). The dual luciferase reporter gene assay results suggested that the luciferase activity was elevated in cells with oe-DLX5 plasmid and c-MYC promoter-WT plasmid (Figure 2G), indicating that DLX5 could combine with the c-MYC promoter. For further verification, expression levels of c-MYC mRNA and protein were determined by RT-qPCR and Western blot analysis in the HFSCs that were treated with oe-DLX5. Treatment with oe-DLX5 plasmid in HFSCs exhibited elevated expression levels of c-MYC mRNA and protein (Figures 2H,I). ChIP experiments verified that c-MYC promoter was bound by DLX5 increased significantly (Figure 2J). The above results indicate that DLX5 can bind to the promoter region of c-MYC and transcriptionally activate c-MYC expression in differentiated HFSCs.

In order to investigate the functional relationship between DLX5 and c-MYC, we transfected HFSCs with oe-DLX5, sh-c-MYC, or both. The results of RT-qPCR and Western blot analysis established that relative to HFSCs with oe-NC and sh-NC, cells transfected with oe-DLX5 and sh-NC showed increased DLX5 mRNA and protein expression levels. However, sh-c-MYC could only inhibit the expression of c-MYC mRNA and protein, and additionally, dual transfection with oe-DLX5 and sh-c-MYC increased DLX5 mRNA and protein expression while reducing c-MYC mRNA and protein expression (Figures 2K,L). Moreover, there was an enhancement in the HFSC proliferation (Figure 2M), and ability to differentiate into sebaceous gland cells and epidermal cells (Figures 2N–P), following transfection with oe-DLX5 while dual transfection with oe-DLX5 and sh-c-MYC induced opposite results. The above results indicate that DLX5 promotes the differentiation of HFSCs by up-regulating the expression of c-MYC in HFSCs.

### c-MYC Binds to the Promoter Region of miR-29c-3p to Inhibit miR-29c-3p Expression, Thereby Promoting HFSC Differentiation

**FIGURE 3:**
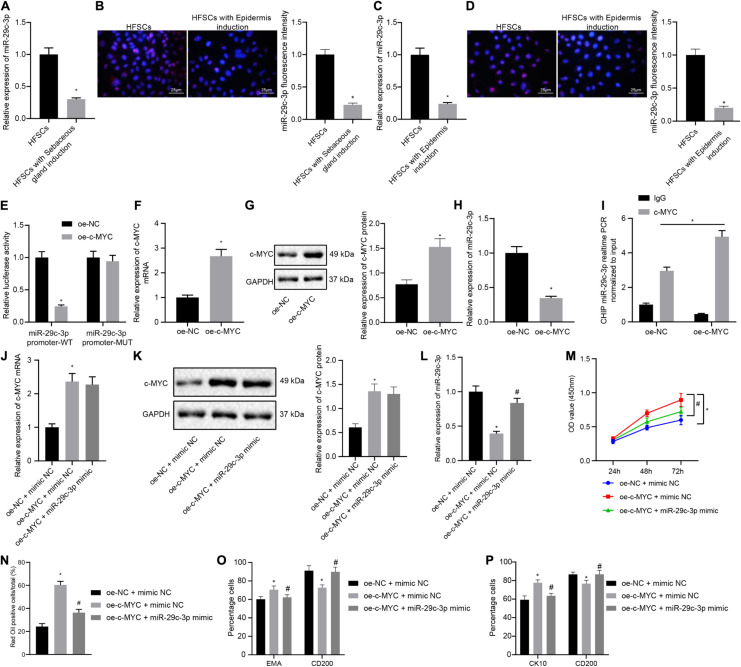
c-MYC promotes the differentiation of HFSCs by down-regulating the expression of miR-29c-3p. **(A)** miR-29c-3p expression in HFSCs after differentiation of HFSCs into sebaceous gland cells as determined by RT-qPCR. *p < 0.05 compared with HFSCs or treatment of mimic NC. **(B)** miR-29c-3p fluorescence intensity in HFSCs after differentiation of HFSCs into sebaceous gland cells as determined by FISH assay. *p < 0.05 compared with HFSCs or treatment of mimic NC. **(C)** miR-29c-3p expression after differentiation of HFSCs into epidermal cells as determined by RT-qPCR. *p < 0.05 compared with HFSCs or treatment of mimic NC. **(D)** miR-29c-3p fluorescence intensity after differentiation of HFSCs into epidermal cells as determined by FISH assay. *p < 0.05 compared with HFSCs or treatment of mimic NC. **(E)** Detection of c-MYC binding to the miR-29c-3p promoter using the dual luciferase reporter gene assay. *p < 0.05 compared with treatment of oe-NC. **(F)** c-MYC mRNA expression in HFSCs treated with oe-c-MYC as determined by RT-qPCR. *p < 0.05 compared with treatment of oe-NC. **(G)** c-MYC protein level in HFSCs treated with oe-c-MYC as determined by Western blot analysis. *p < 0.05 compared with treatment of oe-NC. **(H)** miR-29c-3p expression levels in HFSCs treated with oe-c-MYC as determined by RT-qPCR. *p < 0.05 compared with treatment of oe-NC. **(I)** ChIP analysis of c-MYC binding to the miR-29c-3p promoter. **(J)** c-MYC mRNA expression in HFSCs determined by RT-qPCR. *p < 0.05 compared with treatment of oe-NC + mimic NC. #*p* < 0.05 compared with treatment of oe-c-MYC + mimic NC. **(K)** c-MYC protein level in HFSCs determined by Western blot analysis. *p < 0.05 compared with treatment of oe-NC + mimic NC. #*p* < 0.05 compared with treatment of oe-c-MYC + mimic NC. **(L)** miR-29c-3p expression levels in HFSCs were determined by RT-qPCR. *p < 0.05 compared with treatment of oe-NC + mimic NC. #*p* < 0.05 compared with treatment of oe-c-MYC + mimic NC. **(M)** CCK-8 assay used to detect the proliferation rate of HFSCs. *p < 0.05 compared with treatment of oe-NC + mimic NC. #*p* < 0.05 compared with treatment of oe-c-MYC + mimic NC. **(N)** Oil red O staining before and after the differentiation of HFSCs into sebaceous gland cells. *p < 0.05 compared with treatment of oe-NC + mimic NC. #*p* < 0.05 compared with treatment of oe-c-MYC + mimic NC. **(O)** Detection of EMA and CD200 expression before and after the differentiation of HFSCs into sebaceous gland cells. *p < 0.05 compared with treatment of oe-NC + mimic NC. #*p* < 0.05 compared with treatment of oe-c-MYC + mimic NC. **(P)** Detection of keratin CK10 and CD200 expression before and after the differentiation of HFSCs into epidermal cells. *p < 0.05 compared with treatment of oe-NC + mimic NC. #*p* < 0.05 compared with treatment of oe-c-MYC + mimic NC. The data are expressed as mean ± standard deviation, and comparisons between two groups were analyzed using unpaired *t* test. Comparisons among multiple groups were performed using one-way ANOVA and Tukey’s test. Comparisons among multiple groups at different time points were performed using two-way ANOVA and Bonferroni test. The experiment was repeated three times.

The next step was to probe into whether c-MYC worked through miR-29c-3p in promoting HFSC differentiation. Results of RT-qPCR and FISH suggested that the expression levels of miR-29c-3p were decreased after HFSCs were induced to undergo differentiation into sebaceous gland cells or epidermal cells (Figures 3A–D). The dual luciferase reporter gene assay results suggested that the luciferase activity was decreased in cells transfected with oe-c-MYC plasmid and miR-29c-3p promoter-WT plasmid, indicating that c-MYC bound the miR-29c-3p promoter (Figure 3E). Treatment of HFSCs with oe-c-MYC plasmid resulted in elevated c-MYC mRNA and protein expression levels (Figures 3F,G), along with a reduction in the expression of miR-29c-3p (Figure 3H). Meanwhile, ChIP experimental results confirmed a marked increase in c-MYC binding at the miR-29c-3p promoter (Figure 3I). Together, results suggest that in differentiated HFSCs, c-MYC can bind to the miR-29c-3p promoter region to downregulate the expression of miR-29c-3p.

To characterize the functional relationship between miR-29c-3p and c-MYC, we transfected HFSCs with oe-c-MYC, miR-29c-3p mimic, or both. We found that c-MYC overexpression resulted in enhanced c-MYC mRNA and protein expression (Figures 3J,K), and reduced miR-29c-3p expression (Figure 3L). On the contrary, simultaneous overexpression of miR-29c-3p and c-MYC brought about increased miR-29c-3p and c-MYC expression. Furthermore, as shown in Figures 3M–P, an upward trend was noted in the HFSC viability (Figure 3M), and ability to differentiate into sebaceous gland cells and epidermal cells (Figures 3N–P), following c-MYC overexpression, which was negated by simultaneous overexpression of miR-29c-3p and c-MYC. These results indicate that c-MYC can promote the differentiation of HFSCs by down-regulating the expression of miR-29c-3p in HFSCs.

### miR-29c-3p Targets NSD1 Expression in Differentiated HFSCs

**FIGURE 4:**
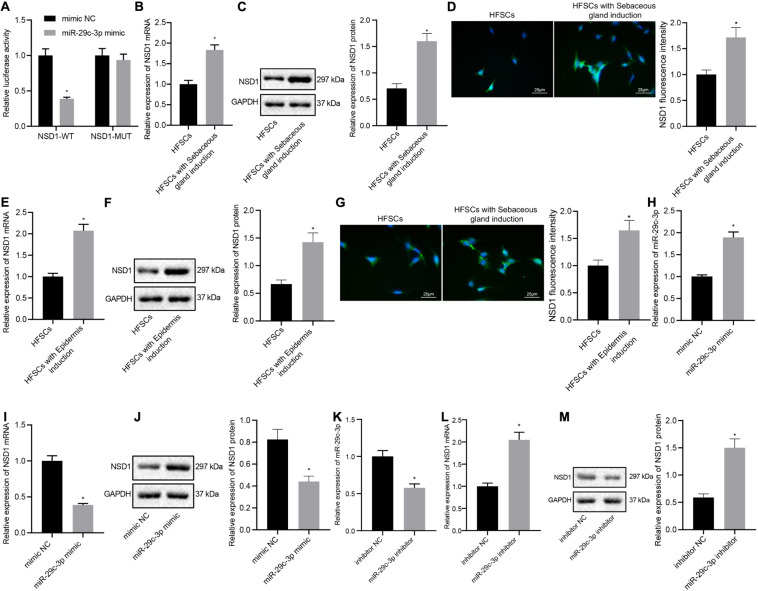
miR-29c-3p targets NSD1 mRNA in differentiated HFSCs. **(A)** Detection of miR-29c-3p binding to NSD1 mRNA using the luciferase activity reporter assay. *p < 0.05 compared with treatment of mimic NC. **(B)** NSD1 mRNA expression after differentiation of HFSCs into sebaceous gland cells as determined by RT-qPCR. *p < 0.05 compared with HFSCs. **(C)** NSD1 protein level after differentiation of HFSCs into sebaceous gland cells as determined by Western blot analysis. *p < 0.05 compared with HFSCs. **(D)** NSD1 fluorescence intensity after differentiation of HFSCs into sebaceous gland cells as determined by immunofluorescence assay (scale bar = 25 μm). *p < 0.05 compared with HFSCs. **(E)** NSD1 mRNA expression after differentiation of HFSCs into epidermal cells as determined by RT-qPCR. *p < 0.05 compared with HFSCs. **(F)** NSD1 protein level after differentiation of HFSCs into epidermal cells as determined by Western blot analysis. *p < 0.05 compared with HFSCs. **(G)** NSD1 fluorescence intensity after differentiation of HFSCs into epidermal cells as determined by immunofluorescence assay (scale bar = 25 μm). *p < 0.05 compared with HFSCs. **(H)** miR-29c-3p expression levels in HFSCs treated with miR-29c-3p mimic as determined by RT-qPCR. **(I)** NSD1 mRNA expression in HFSCs treated with miR-29c-3p mimic as determined by RT-qPCR. *p < 0.05 compared with treatment of mimic NC. **(J)** NSD1 protein level in HFSCs treated with miR-29c-3p mimic as determined by Western blot analysis. *p < 0.05 compared with treatment of mimic NC. **(K)** miR-29c-3p expression levels in HFSCs treated with miR-29c-3p inhibitor (plasmid suppressing the expression of miR-29c-3p) as determined by RT-qPCR. **(L)** NSD1 mRNA protein expression in HFSCs treated with miR-29c-3p inhibitor as determined by RT-qPCR. *p < 0.05 compared with treatment of mimic NC. **(M)** NSD1 protein level in HFSCs treated with miR-29c-3p inhibitor as determined by Western blot analysis. *p < 0.05 compared with treatment of mimic NC. The data are expressed as mean ± standard deviation, and comparisons between two groups were analyzed by unpaired *t* test. The experiment was repeated three times.

Next, we investigated whether miR-29c-3p regulated NSD1 during HFSC differentiation. The results from the dual luciferase reporter gene assay illustrated that treatment with miR-29c-3p mimic plasmids resulted in decreased luciferase activity of NSD1-WT, indicating that NSD1 mRNA was a target of miR-29c-3p (Figure 4A). In addition, we found that NSD1 expression was increased after differentiation of HFSCs into sebaceous gland cells or epidermal cells (Figures 4B–G). Overexpression of miR-29c-3p in HFSCs enhanced miR-29c-3p expression (Figure 4H) and reduced NSD1 mRNA and protein expression (Figures 4I,J). Downregulation of miR-29c-3p in HFSCs led to a decline in miR-29c-3p expression (Figure 4K), and enhanced NSD1 mRNA and protein expression (Figures 4L,M). These data suggest that miR-29c-3p suppresses NSD1 expression in differentiated HFSCs.

### Overexpression of NSD1 Reverses miR-29c-3p-Mediated Inhibition of HFSC Differentiation

**FIGURE 5:**
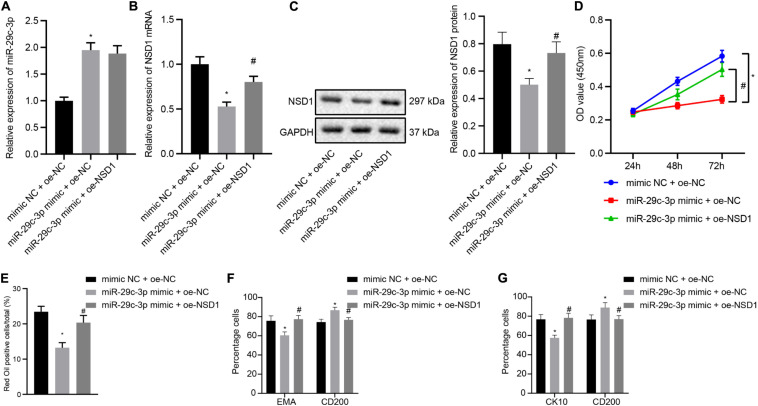
NSD1 overexpression reverses the effects of miR-29c-3p to promote HFSC differentiation. **(A)** miR-29c-3p expression in HFSCs was determined by RT-qPCR. **(B)** NSD1 mRNA expression in HFSCs determined by RT-qPCR. **(C)**. NSD1 protein level in HFSCs determined by Western blot analysis. **(D)** CCK-8 assay used to detect the proliferation rate of HFSCs. **(E)** Oil red O staining before and after differentiation of HFSCs into sebaceous gland cells. **(F)** Detection of EMA and CD200 expression before and after the differentiation of HFSCs into sebaceous gland cells using flow cytometry. **(G)** The detection of keratin CK10 and CD200 expression before and after the differentiation of HFSCs into epidermal cells using flow cytometry. *p < 0.05 compared with treatment of mimic NC + oe-NC. #*p* < 0.05 compared with treatment of miR-29c-3p mimic + oe-NC. The data are expressed as mean ± standard deviation. Comparisons among multiple groups were performed using one-way ANOVA and Tukey’s test. Comparisons among multiple groups at different time points were performed using two-way ANOVA and Bonferroni test. The experiment was repeated three times.

In this part, we attempt to elucidate the functional relationship between miR-29c-3p and NSD1. HFSCs were transfected with miR-29c-3p mimic, oe-NSD1 or both. At first, we found that treatment with exogenous miR-29c-3p led to upregulated miR-29c-3p expression (Figure 5A) and downregulated NSD1 mRNA and protein expression (Figures 5B,C). Their expression was observed to be both increased following concomitant overexpression of miR-29c-3p and NSD1. In addition, miR-29c-3p mimic reduced HFSC viability (Figure 5D), and ability to differentiate into sebaceous gland cells and epidermal cells (Figures 5E–G) while simultaneous overexpression of miR-29c-3p and c-MYC led to opposite results. These results indicate that in HFSCs, overexpression of NSD1 can reverse the role of miR-29c-3p and promote HFSC differentiation.

### DLX5 Promotes HFSC Differentiation by Regulating the c-MYC/miR-29c-3p/NSD1 Axis

**FIGURE 6:**
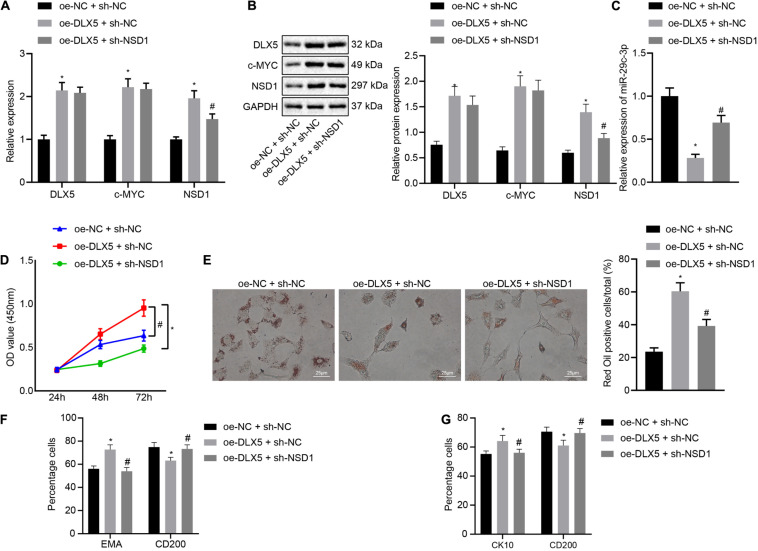
DLX5 induces HFSC differentiation by regulating the c-MYC/miR-29c-3p/NSD1 axis. **(A)** DLX5, NSD1, and c-MYC mRNA expressions in HFSCs as determined by RT-qPCR. **(B)** DLX5, NSD1, and c-MYC protein levels in HFSCs as determined by Western blot analysis. **(C)** miR-29c-3p expression levels in HFSCs were measured using RT-qPCR. **(D)** CCK-8 assay used to detect the proliferation rates in HFSCs. **(E)** Oil red O staining before and after differentiation of HFSCs into sebaceous gland cells (scale bar = 25 μm). **(F)** Detection of EMA and CD200 expression before and after differentiation of HFSCs into sebaceous gland cells using flow cytometry. **(G)** Detection of keratin CK10 and CD200 expression before and after the differentiation of HFSCs into epidermal cells using flow cytometry. *p < 0.05 compared with treatment of oe-NC + sh-NC; #*p* < 0.05 compared with treatment of oe-DLX5 + sh-NC. The data were expressed as mean ± standard deviation. Comparisons among multiple groups were performed using one-way ANOVA and Tukey’s test. Comparisons among multiple groups at different time points were performed using two-way ANOVA and Bonferroni test. The experiment was repeated three times.

Finally, we investigated the role of the DLX5/c-MYC/miR-29c-3p/NSD1 regulatory axis during HFSC differentiation. To address this question, we introduced oe-DLX5, sh-NSD1 or both into HFSCs. Knockdown of NSD1 increased the mRNA transcription and protein levels of DLX5, c-MYC, and NSD1 (Figures 6A,B), while decreasing the expression of miR-29c-3p (Figure 6C). Dual transfection with oe-DLX5 and sh-NSD1 led to an enhancement in the mRNA and protein expression of DLX5, c-MYC, and NSD1 as well as miR-29c-3p expression. Moreover, oe-DLX5 increased HFSC viability (Figure 6D), and ability to differentiate into sebaceous gland cells and epidermal cells (Figures 6E–G) while co-transfection with oe-DLX5 and sh-NSD1 led to opposite results. These results suggest that DLX5 induces HFSC differentiation by regulating the c-MYC/miR-29c-3p/NSD1 axis.

### DLX5 Promotes Hair Follicle Regeneration by Regulating the c-MYC/miR-29c-3p/NSD1 Axis

**FIGURE 7:**
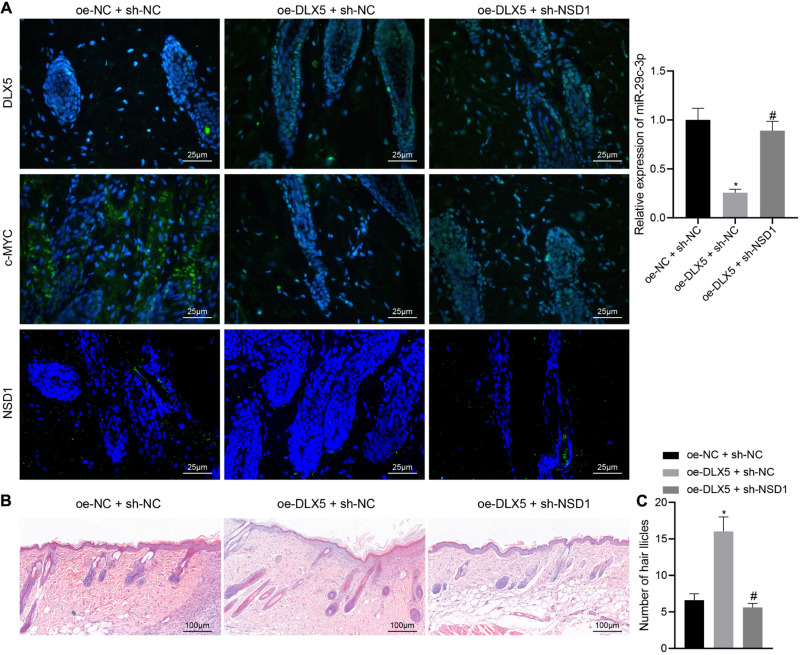
DLX5 facilitates the hair follicle regeneration by regulating the c-MYC/miR-29c-3p/NSD1 signaling axis. **(A)** Expression of DLX5, c-MYC, and NSD1 determined by immunofluorescence assay in the back skin tissues of mice treated with oe-DLX5, sh-NSD1 or both (left panel); RT-qPCR analysis of miR-29c-3p expression in different group of the skin (right panel). **(B)** HE staining of back skin tissues of mice treated with oe-DLX5, sh-NSD1 or both after transplantation for 3 weeks (× 400). **(C)** Counting of the hair follicle like structure of back skin tissues of mice treated with oe-DLX5, sh-NSD1 or both after transplantation for 3 weeks. *p < 0.05 compared with treatment of oe-NC + sh-NC; #*p* < 0.05 compared with treatment of oe-DLX5 + sh-NC. The data were expressed as mean ± standard deviation. Comparisons among multiple groups were performed using one-way ANOVA and Tukey’s test. *N* = 5 for mice following each treatment.

Hair follicle stem cells were transfected with oe-DLX5 or in combination with sh-NSD1. A 2.5 mm full-thickness skin wound was made on the back of nude mice, and the HFSCs were mixed with dermal papilla cells and transplanted into the skin wound. One week later, the wound healed. Three weeks after transplantation, the mice were euthanized killed and the expression of DLX5, c-MYC, and NSD1 in the back skin tissues was detected by immunofluorescence staining. The confocal images indicated that DLX5 overexpression increased the expression of DLX5, c-MYC, and NSD1, while no changes were observed in the expression of DLX5 and c-MYC in addition to inhibited NSD1 expression (Figure 7A left panel) upon treatment with both oe-DLX5 and sh-NSD1. Moreover, as RT-qPCR analysis revealed, the miR-29c-3p expression was reduced in oe-DLX5 + sh-NC group compared with oe-NC + sh-NC (Figure 7A right panel). HE staining showed hair follicle like structure under the skin (Figure 7B). Additionally, hair follicle like structure was increased in response to overexpression of DLX5 while it was decreased upon treatment with both oe-DLX5 and sh-NSD1 (Figure 7C). These results indicate that DLX5 promotes hair follicle regeneration by regulating the c-MYC/miR-29c-3p/NSD1 signaling axis.

## Discussion

Due in part to the complex interaction among dermal cells, epithelial cell, and other cell types, the HFSCs with differentiation potential have been regarded to have prospective therapeutic applications (Waters et al., 2007). In this study, we sought to elucidate the downstream molecular mechanism of transcription factor DLX5 in HFSC differentiation. Collectively, our study suggested that DLX5 could bind to the promoter region of c-MYC, a biological macromolecule downstream of the Wnt/β-catenin signaling pathway, transcriptionally activating its expression. Meanwhile, c-MYC could bind to the promoter region of miR-29c-3p to inhibit its expression, causing the upregulation of miR-29c-3p target NSD1, thereby promoting HFSC differentiation.

We found that DLX5 expression was elevated in differentiated HFSCs and that increased DLX5 transcriptionally activated c-MYC expression. DLX5 was previously shown to act as a transcriptional repressor of downstream target gene expression, and the protein-protein interactions are significant for specification of the transcriptional activities of DLX5 (Lee et al., 2013). Previous studies have reported that DLX5 is upregulated in human lymphomas and non-small cell lung cancer, where it could bind to the MYC promoter to activate its transcription (Xu and Testa, 2009; Sun et al., 2020). Furthermore, it has been shown that MYC superfamily members are essential for the epithelial cell differentiation of the hair follicle, which may play a role in the fate determination of stem cells (Bull et al., 2001; Flores et al., 2017; Shen et al., 2017; Zhao et al., 2020). Interestingly, we also found that the transcription factor DLX5 could bind to the promoter region of c-MYC. The Wnt/β-catenin pathway is an important pathway controlling cell proliferation and differentiation. In fact, β-catenin plays a vital role in HFSC differentiation, and could induce differentiation of HFSC into transit-amplifying cells via c-MYC activation (Shen et al., 2017). The expression of the distal-less homeobox gene family including DLX2, DLX3, DLX5, and DLX6 can be increased by KDM4B. It has been reported that DLX2, DLX3, and DLX5 all take part in osteoblast differentiation (Zeng et al., 2020). DLX2 is a transcription factor which can counteract TGF-β induced cell-cycle arrest and apoptosis (Lee et al., 2015). DLX2 is also involved in glycolytic switch, Wnt- and TGF-β-induced epithelial-mesenchymal transition (EMT) and mitochondrial repression (Westendorf et al., 2004). It has reported that DLX2 binds to WNT1, then up-regulates Wnt1 transcription and activated Wnt/β-catenin signal pathway, suggesting that DLX2 is an activator for osteogenic differentiation (Zeng et al., 2020). Zhang S. et al. (2012) have found that c-MYC downregulation contributed and induced teratocarcinoma cell differentiation caused by activation of Wnt/β-catenin signaling. Furthermore, another study has demonstrated that knockdown of c-MYC attenuated Wnt/β-catenin-mediated induction of human sertoli cell proliferation (Li et al., 2012).

The present study suggested that c-MYC bound to miR-29c-3p promoter region suppressing its expression and that NSD1 negated the inhibitory effect of miR-29c-3p on HFSC differentiation. In addition, c-MYC is associated with the aggressive nature of cancers and modulates the expression of several miRNAs in various malignancies (Chen et al., 2018). One study revealed that miR-29a appears to regulate its effects on hematopoietic stem cell (HSCs) function through modulating the DNA methyltransferase enzyme activity (Hu et al., 2015). Upregulation of miR-29a induces cardiac stem cells to differentiate into cardiomyocytes by suppressing endogenous Wnt/β-catenin (De Pauw et al., 2013). Mott et al. (2010) suggested that the pro- or anti-apoptotic functions of c-MYC may depend on the integration of competing signals, including miR-29 repression. As previously described, Neurotensin signaling stimulates glioblastoma cell proliferation by upregulating c-Myc and inhibiting miR-29b-1 and miR-129-3p (Ouyang et al., 2016). Another study suggested that c-MYC repressed miR-29b-1 expression in the proliferation of glioblastoma cells (Ouyang et al., 2016). Similar to our study, a recent article has indicated that DLX5 was targeted by miR-339-5p, which may directly regulate HFSC differentiation (Li et al., 2018). NSD family members have been found in human malignancies, and shown to play a vital role in cell growth and differentiation (Bennett et al., 2017; Rayasam et al., 2003). A prior article has demonstrated that NSD1 could be regarded as a nuclear localized basic transcriptional factor as well as a bifunctional transcriptional regulator (Kurotaki et al., 2001). Nevertheless, the binding of miR-29c-3p and NSD1 needs further verification.

**FIGURE 8:**
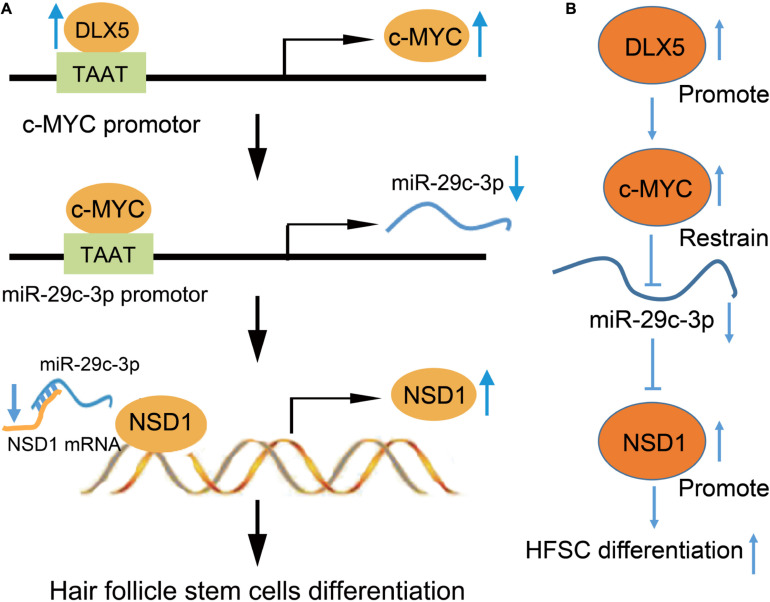
Mechanistic diagrams. **(A)** DLX5 binds to the promoter region of c-MYC, leading to c-MYC transcriptional activation to increase its expression. The increased expression of c-MYC enhances the binding of c-MYC in the promoter region of miR-29c-3p, which inhibits the transcription of miR-29c-3p. The decreased expression of miR-29c-3p weakened the inhibitory effect of miR-29c-3p on NSD1-mRNA. Therefore, the increased expression of NSD1 promotes the differentiation of HFSCs. **(B)** DLX5 promotes the transcription of c-MYC and c-MYC restrains the transcription of miR-29c-3p. The downregulated expression of miR-29c-3p reduced the inhibition of miR-29c-3p on NSD1-mRNA. Therefore, the increased expression of NSD1 promotes the differentiation of HFSCs. Therefore, our results showed the promoting effect of the DLX5/c-MYC/miR-29c-3p/NSD1 axis on HFSC differentiation, that DLX5 promotes the transcriptional expression of c-MYC to inhibit the expression of miR-29c-3p, thus reducing the inhibitory effect of miR-29c-3p on NSD1. Considering that NSD1 promotes HFSC differentiation, DLX5 increases the expression of NSD1 to promote HFSC differentiation.

Taken together, these results support the hypothesis that DLX5 promotes HFSC differentiation via the regulation of the c-MYC/miR-29c-3p/NSD1 axis (Figure 8). In addition, this study provides a novel pathway for the further exploration of HFSC differentiation.

## Data Availability Statement

The original contributions presented in the study are included in the article/Supplementary Material, further inquiries can be directed to the corresponding author.

## Ethics Statement

The animal study was reviewed and approved by the Animal Ethics Committee of First Affiliated Hospital of Guangxi Medical University.

## Author Contributions

BL, JZ, and GY: study concept and design. ML, GL, and YY: data acquisition and analysis. BL, DH, and SL: manuscript writing. All authors have read and approved the final submitted manuscript.

## Conflict of Interest

The authors declare that the research was conducted in the absence of any commercial or financial relationships that could be construed as a potential conflict of interest.
