# Supplementary material for: Transcription Factor DLX5 Promotes Hair Follicle Stem Cell Differentiation by Regulating the c-MYC/microRNA-29c-3p/NSD1 Axis
Source: Front Cell Dev Biol. 2021 Jul 15;9:554831. doi: 10.3389/fcell.2021.554831 (PMC8319474; doi:10.3389/fcell.2021.554831)
Supplement: Supplementary file 1 [file Table_1.DOCX]

**Table S1** Primer sequence

| Name | Sequence |
| --- | --- |
| DLX5-F | 5′-CCGCTTTACAGAGAAGGTTTCA-3′ |
| DLX5-R | 5′-TCTTCTTGATCTTGGATCTTTTGTT-3′ |
| c-MYC-F | 5′-CGTCCTCGGATTCTCTGCTC-3′ |
| c-MYC-R | 5′-GCTGGTGCATTTTCGGTTGT-3′ |
| miR-29c-3p-F | 5′-GTCCGCTAGCACCATTTGAAATCGGTTA-3′ |
| miR-29c-3p-R | 5′-GTGCGTGTCGTGGAGTC-3′ |
| NSD1-F | 5′-ACCTGACAGAGCCTCTCCAA-3′ |
| NSD1-R | 5′-GCTGGAGTTTTCTCCACTGC-3′ |
| U6-F | 5′-GTCCGCGTGCTCGCTTCGGCAGC-3′ |
| U6-R | 5′-GTGCGTGTCGTGGAGTC-3′ |
| GAPDH-F | 5′-CAAGAAGGTGGTGAAGCAGG-3′ |
| GAPDH-R | 5′-CCACCCTGTTGCTGTAGCC-3′ |

Note: F, forward; R, reverse; GAPDH, glyceraldehyde-3-phosphate dehydrogenase.
